# Results from a national treatment database – does it matter which ART combination is prescribed in the real world?

**DOI:** 10.7448/IAS.17.4.19614

**Published:** 2014-11-02

**Authors:** Gordon Scott, Lesley Wallace

**Affiliations:** 1 Sexual Health, Chalmers Sexual Health Centre, Edinburgh, UK; 2 Epidemiology, Health Protection Scotland, Glasgow , UK

## Abstract

**Introduction:**

Clinical trials frequently show differences in viral load (VL) response between antiretroviral therapy (ART) regimes. Patterns of prescribing vary from country to country (Mocroft et al. Infection 2014 Jun 6 [epub ahead of print]), and are likely to vary between individual clinics. Scotland has a national database that records VL results and specific ART regimes for every patient under care, thus allowing different prescribing patterns between clinical centres to be monitored. Does this reveal any difference in achievement of undetectable VL?

**Materials and Methods:**

We interrogated the database held by Health Protection Scotland (HPS) that contains a record of every VL result matched against prescribed ART. Results were censored at the end of December 2013 and are based on the latest attendance of patients who have been under monitoring for at least six months. For simplicity, we have broken the results into class of drug rather than individual drugs for example, nucleoside reverse transcriptase inhibitor (NRTI) rather than lamivudine, abacavir etc. The data were analyzed using univariate Poisson regression.

**Results:**

The anonymized records of 3302 individual patients who attended in 11 separate regions were scrutinized. Sixty-eight different combinations of antiretroviral regimes were identified. The prescribing patterns for the five most frequently prescribed regimes in the four largest clinics are shown in Table 1, along with the overall percentage of patients with undetectable VL. A higher proportion of patients in Scotland who are prescribed regimes of NRTI×2 or NRTI/NtRTI plus PI have detectable VL but this is not statistically significant. Although the percentage of patients with VL<50 varies between regions 1 and 4 versus regions 2 and 3, this is also not statistically significant.

**Conclusions:**

Overall, a high proportion of Scottish patients on ART have undetectable VL. Patterns of ART prescribing in Scotland do vary by region but there are no significant differences in outcome with regard to undetectable VL. There is a non-significant trend which may be accounted for by differing levels of PI prescribing.

**Table 1:** Regional patterns of prescribing

	NRTI×2 +PI (%)	NRTI×2 +NNRTI (%)	NRTI/NtRTI/PI (%)	NRTI/NtRTI/NNRTI (%)	NRTI/NtRTI/II (%)	% VL < 50
Region 1	7	6	16	61	1	91
Region 2	6	14	9	35	5	96
Region 3	3	11	5	38	5	96
Region 4	4	4	25	47	8	87
Scottish total	5	11	11	41	5	95

